# ESBL production and carbapenem resistance increased the secondary bloodstream infection rates in intensive care units in Turkey, 2014–2019

**DOI:** 10.3205/dgkh000408

**Published:** 2022-04-11

**Authors:** Can Huseyin Hekimoglu, Serap Suzuk Yildiz, Selda Sahan, Esen Batir, Emine Yildirim Gozel, Dilek Altun, Gulen Pehlivanturk, Muhammet Comce, Fatih Kara

**Affiliations:** 1 Ministry of Health, General Directorate of Public Health, Department of Communicable Diseases, Ankara, Turkey; 2 Ministry of Health, General Directorate of Public Health, Department of Microbiology Reference Laboratory and Biological Products, Ankara, Turkey; 3 Ministry of Health, General Directorate of Public Health, Department of Tuberculosis, Ankara, Turkey; 4 Ministry of Health, General Directorate of Public Health, Ankara, Turkey

**Keywords:** secondary bloodstream infection, catheter-associated urinary tract infection, ventilator-associated pneumonia, ventilator-associated event, carbapenem resistance, extended spectrum beta-lactamase

## Abstract

**Aim:**

Secondary bloodstream infections (SBSIs) are caused by another infection and differ from primary bloodstream infections (PBSIs) in terms of prevention and treatment strategies. The aim of this study was to determine the risk factors for bloodstream infections which were secondary to the most common healthcare-associated infections caused by the most common microorganisms in intensive care units (ICUs) and to examine whether extended-spectrum beta lactamase (ESBL) production and carbapenem resistance is related to the higher risk or not.

**Methods:**

The study population consisted of patients in ICUs with ventilator-associated pneumonia (VAP), ventilator-associated event (VAE) or catheter-associated urinary tract infection (CAUTI) caused by *E. coli, K. pneumoniae, P. aeruginosa* or *A. baumannii* between 2014 and 2019. The data were obtained through the National Healthcare-associated Infections Surveillance Network. Multivariate logistic regression analysis was performed separately for VAP/VAE and CAUTI to determine the risk factors for the development of SBSI.

**Results:**

Microorganism, ICU type, bed capasity and carbapenem resistance were found to be risk factors for SBSI for both types of infection. For VAPs/VAEs, female gender and hospital type were also identified as risk factors. The highest risk was in *K.pneumoniae* and in emergency ICUs. Among the hospitals, the highest risk in VAPs/VAEs was found in government education and research hospitals. ESBL production for *K*. *pneumoniae* and *E. coli* increased the risk in patients with VAP/VAE; however, it did not increase in patients with CAUTI.

**Discussion:**

By using the risk factors, it may be possible to recognize SBSIs earlier, especially in patients with CAUTIs or VAPs/VAEs caused by carbapenem-resistant or ESBL-producing *K. pneumoniae*.

## Zusammenfassung

**Hintergrund:**

Bei der sekundären Blutstrominfektion (SBI) gelangt der Erreger von einem anderen Infektionsort in die Blutbahn; daher unterscheidet sich die Präventions- und Behandlungsstrategie von der primären Blutstrominfektion (PBI). Ziel der Studie war es, Risikofaktoren für SBI, die zu den häufigsten nosokomialen Infektionen gehören, zu bestimmen und zu untersuchen, ob Extended-Spectrum-Beta-Laktamase (ESBL) Bildner und Carbapenemresistenz mit einem höheren Risiko für SBI assoziiert sind.

**Methode:**

Die Studienpopulation bestand aus Patienten auf Intensivstationen mit Beatmungs-assoziierter Pneumonie (VAP), Beatmungs-assoziiertem Ereignis (VAE) oder Katheter-assoziierter Harnweginfektion (CAUTI), die durch *E. coli*, *K. pneumoniae*, *P. aeruginosa* oder *A. baumannii* zwischen 2014–2019 verursacht wurden. Die Daten wurden über das National Healthcare-associated Infections Surveillance Network erhalten. Die multivariate logistische Regressionsanalyse wurde getrennt für VAP/VAE und HWI durchgeführt, um die Risikofaktoren für die Entwicklung einer SBI zu bestimmen.

**Ergebnisse:**

Mikroorganismen, Art der ITS, Bettenkapazität und Carbapenemresistenz wurden als Risikofaktoren für SBI für beide Infektionsarten gefunden. Für VAP/VAE wurden zusätzlich weibliches Geschlecht und der Krankenhaustyp als Risikofaktoren identifiziert. Das höchste Risiko bestand bei *K. pneumoniae* und auf Notfall-Intensivstationen. Unter den Krankenhäusern wurde das höchste Risiko für VAP/VAE in staatlichen Lehr- und Forschungskrankenhäusern festgestellt. Die ESBL-Bildung bei *K. pneumoniae* und *E. coli* erhöhte das Risiko einer SBI bei Patienten für VAP/VAE, nicht jedoch bei Patienten mit HWI.

**Schlussfolgerung:**

Durch die Kenntnis von Risikofaktoren können SBI möglicherweise früher erkannt werden, insbesondere bei Patienten mit HWI oder VAP/VAE, die durch Carbapenem-resistente oder ESBL-produzierende *K. pneumoniae* verursacht werden.

## Introduction

Secondary bloodstream infections (SBSIs) are defined as infections caused by a microorganism that passes into blood from a primary infection site [1], [2]. SBSIs, which are associated with increased mortality, prolonged hospital stay, and higher costs, are preventable with early treatment of the primary infection. It is difficult to talk about its general frequency, since there are few studies on the epidemiology of SBSI in the literature, and none on a large scale [2].

According to the national surveillance reports in Turkey, the SBSI rate among all heathcare-associated infections was 7.0% in 2020, while its ratio among all type of BSIs was 14.4%. The infection type with the highest SBSI rate was soft tissue infections (16.1%); however, the highest number of SBSIs was developed due to different kinds of pneumonia and urinary tract infections, mostly invasive device-associated. The most common agents causing ventilator-associated pneumonia (VAPs) are *Acinetobacter baumannii*, *Klebsiella pneumoniae* and *Pseudomonas aeruginosa*, and the most common pathogens causing catheter-related urinary tract infections (CAUTIs) are *K. pneumoniae*, *Escherichia. coli* and *P. aeruginosa* [3].

Secondary BSIs are not directly related to the central line or any other invasive device, and preventive strategies for SBSIs differ from those fro primary bloodstream infections (PBSIs). Therefore, it is of great importance to make a correct distinction between PBSIs and SBSIs. The source of infection should be investigated by recording the symptoms and collecting other types of cultures, including blood cultures, from patients in whom microorganisms were detected with primary site-specific infections [4]. Therefore, it would be useful to determine the factors (hospitals, intensive care unit wards, patients, microorganisms and resistance patterns) which affected the development of SBSI. In this study, we aimed to determine the risk factors for SBSIs that developed from VAPs/VAEs and CAUTIs and were caused by the most common microorganisms in intensive care units (ICUs). We also examined whether extended spectrum beta lactamase (ESBL) production and carbapenem resistance is related to higher risk or not.

## Methods

### Study population

The study population consisted of patients hospitalized in ICUs (except neonatal intensive care units) and diagnosed with VAP, VAE or CAUTI caused by *E. coli, K. pneumoniae, P. aeruginosa* or *A. baumannii* in Turkey between 2014 and 2019. All data were obtained through the National Healthcare-associated Infections Surveillance Network (USHIESA). It is mandatory to enter data on active, prospective, invasive device-associated infection surveillance by all hospitals according to the National Surveillance Standards [5]. VAP, ventilator-associated events (VAE) and CAUTI caused by *E. coli, K. pneumoniae, P. aeruginosa* and *A. baumannii* were selected for the study, since they are the most common infections and agents involved in SBSIs in ICUs in Turkey [3].

### Variables

The dependent variable was the development of SBSI. SBSI was diagnosed when at least one microorganism matching the agent at the original infection site was isolated in a blood culture collected within the SBSI attribution period, or if the microorganism isolated from the blood culture collected within the infection period is one of the site-specific infection diagnostic criteria according to the National Surveillance Diagnostic Guideline [6].

The independent variables were infection type (VAP/VAE or CAUTI), microorganism (*E. coli, K. pneumoniae, P. aeruginosa, A. baumannii*), sex (female/male), hospital type (private hospitals, government hospitals, training and research hospitals and university hospitals), bed capacity, ICU type (pediatric ICUs, emergency ICUs, surgical ICUs, internal ICUs, mixed ICUs and anesthesiology and reanimation ICUs), carbapenem susceptibility (susceptible/resistant) and ESBL (ESBL producing, non-ESBL producing). In Turkey, monitored the VAE or VAP surveillance is mandatory in all ICUs, but VAE surveillance has been optional since 2015. Therefore, VAP and VAE were considered together in the study. Only possible and probable VAPs were included in the study, since no microorganism was recorded for a ventilator-associated condition or an infection-related ventilator-associated complication within the scope of VAE surveillance.

Bed capacity was examined both as a continuous variable (the number of hospital beds) and as a binary variable (<750 beds ≥750 beds). Carbapenem resistance and ESBL production of the microorganisms included in the study are among the mandatory data that should be collected by all hospitals in Turkey. All microbiology laboratories have followed EUCAST (The European Committee on Antimicrobial Susceptibility Testing) standards since 2014. 

### Statistical analysis

We summarized categorical variables with numbers (n), percentages (%) and 95% confidence intervals (CI), and used the chi-squared test for comparisons. The change over the years was examined with the chi-squared test for trends. Bed capacity was summarized with mean and standard deviation, and Student’s t-test for independent samples was used for comparisons.

The SBSI rate was calculated with the formula of SBSI number/total number of infections x100, and expresses what percentage of infections develop SBSI. Odds ratios (ORs) and 95% CIs were calculated to determine the risk of developing SBSI. To determine the risk factors, multivariate logistic regression analysis with stepwise forward variable selection was performed separately for VAP/VAE and CAUTI. To determine the adjusted effect of ESBL production for *E. coli* and *K. pneumoniae*, the adjusted ORs for the identified risk factors were calculated using a multivariate logistic regression analysis with the enter selection method separately for VAP/VAE and CAUTI. Data were analyzed using SPSS version 20.0 (SPSS IBM; Armonk, NY, USA). Statistical significance was set at 0.05 for all statistical tests.

## Results

A total of 71,955 infections, including 32.4% (n=23,281) CAUTI, 59.8% (n=43,018) VAP and 7.9% (n=5,656) VAE, were examined. The SBSI rate was found to be

- 11.7% (95%CI=11.4–11.9) overall and
- 11.2% (95%CI=10.8–11.6), and
- 11.9% (95%CI=11.6–12.2) for VAPs/VAEs and CAUTIs, respectively (p=0.004).

*A. baumannii* was the major agent, with a rate of 40.5% among the microorganisms, and 70.2% (95%CI=69.7%–70.8%) of them were resistant to carbepenem. Carbapenem resistance was higher in *K. pneumoniae* isolates (42.3%; 95%CI=41.5%–43.1%) than in *P. aeruginosa* isolates (35.4%; 95%CI=34.7%–36.2%), while it was the lowest in *E. coli* isolates (7.8%; 95%CI=7.3%–8.3%). ESBL production was higher in *K. pneumoniae* isolates (47.4%; 95%CI=46.%–48.2%) than in *E. coli* isolates (43.0%; 95%CI=42.1%–43.9%) (p<0.001). The proportions of both ESBL-producing and carbapenem-resistant isolates were 5.1% (95%CI=4.7%–5.5%) and 26.6% (95%CI=25.9%–27.3%) for *E. coli* and *K. pneumoniae*, respectively (p<0.001). 

### VAPs/VAEs

Among the VAPs/VAEs, the most common microorganism was *A. baumannii*, with a rate of 53.0%, followed by *P. aeruginosa* (23.4%), *K. pneumoniae* (17.5%) and *E. coli* (6.0%), respectively. Carbapenem resistance was the highest in *A. baumannii* isolates (70.3%; 95%CI=69.7%–70.9%) vs the other isolates, followed by *K. pneumoniae* (43.0%; 95%CI=41.7%–43.8%), *P. aeruginosa* (36.9%; 95%CI=36.1%–37.8%) and finally *E. coli* with the lowest rate of carbapanem resistance (11.5%; 95%CI=10.4%–12.7%). ESBL production was similar between *K. pneumoniae* (45.7%; 95%CI=44.6%–46.8%) and *E. coli* isolates (44.3%; 95%CI=42.5%–46.1%) (p=0.178).

While the highest overall SBSI rate from VAPs/VAEs was caused by *K. pneumoniae* with a rate of 15.0%, the lowest rate (9.5%) was found in VAPs/VAEs caused by *P. aeruginosa*. The rates for these two microorganisms were significantly different when compared with the others (p<0.05). However, the overall rate was similar in the VAPs/VAEs in which *E. coli* and *A. baumannii* were the causative agents (Table 1 (Tab. 1)).

There was a decreasing trend in the number of VAPs/VAEs over the years, while an increase was observed in SBSI rates. When analyzed according to microorganisms, an increasing trend in SBSI was detected, except for *E. coli* (Table 1 (Tab. 1)). 

**Figure 1:**
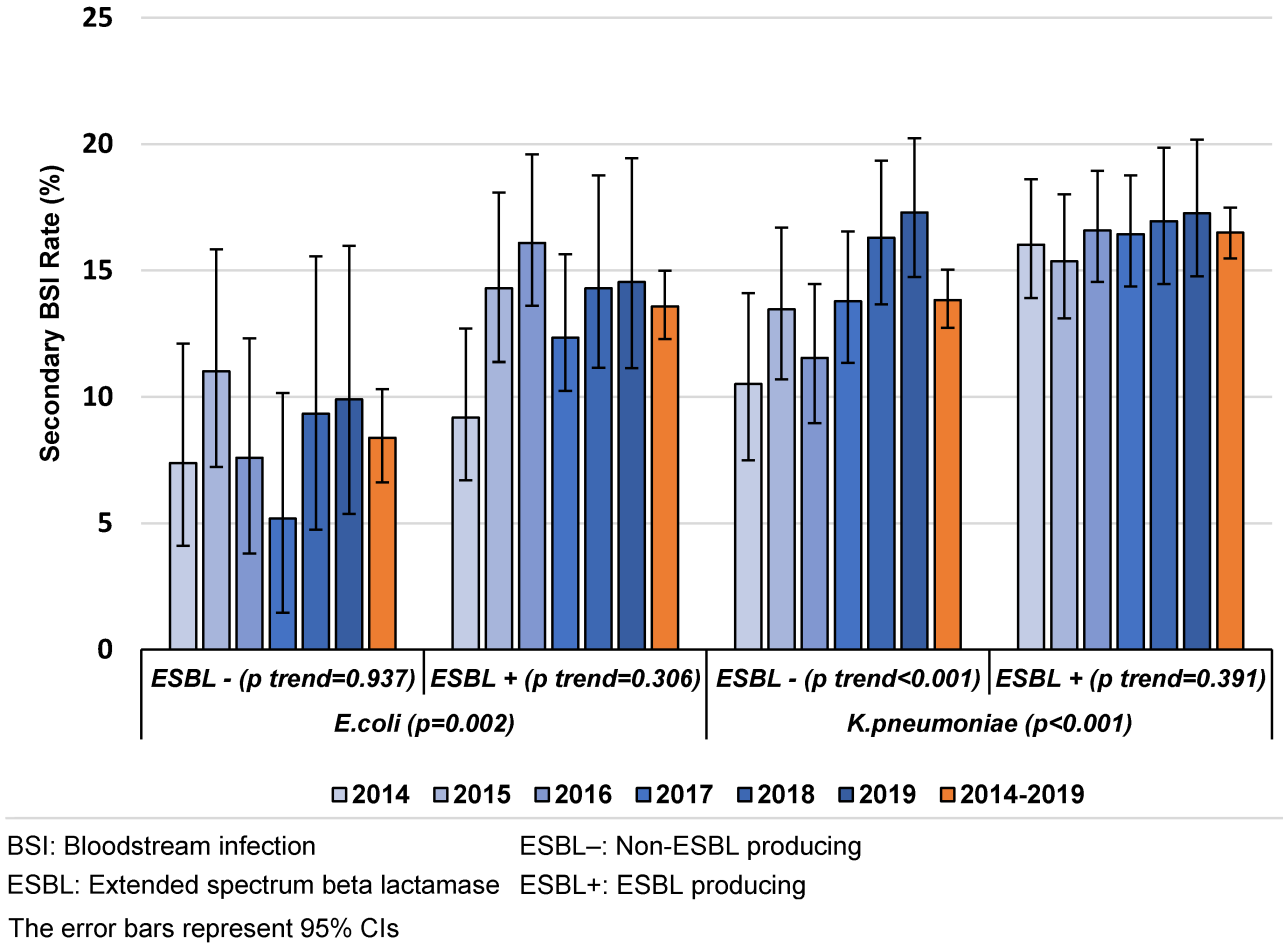
The distribution of secondary BSI rates in VAPs/VAEs caused by *E. coli* and *K. pneumoniae* by ESBL production and year, Turkey, 2014–2019 (n=11,453)

Production of ESBL was found to be a risk factor for SBSI caused both by *K. pneumoniae* and *E. coli*. While ESBL-producing and non-ESBL-producing *K. pneumoniae* were associated with a higher SBSI rate than ESBL-producing and non-ESBL-producing *E. coli* (p<0.001 and p<0.001, respectively) in all years, an increasing trend of the SBSI rates was observed with non-ESBL-producing *K. pneumoniae* (p<0.001) (Figure 1 (Fig. 1)).

**Figure 2:**
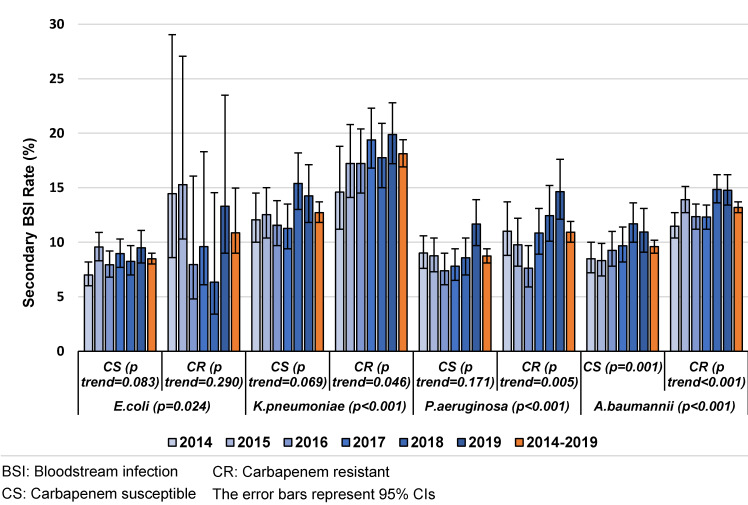
The distribution of secondary BSI rates in VAPs/VAEs caused by different microorganisms, carbapenem resistance and years, Turkey, 2014–2019 (n=54,560)

Carbapenem resistance was found to be another risk factor for SBSI in VAPs/VAEs caused by one of the microorganisms in the study. While the SBSI rate tended to increase with carbapenem-resistant *K. pneumoniae* and *P. aeruginosa* during the years of observation, there was an also increasing trend in both carbepenem-resistant and carbapenem-susceptible *A. baumannii* isolates (Figure 2 (Fig. 2)). In 2019, the rate reached 20% in the presence of carbapenem resistance in *K. pneumoniae*, and the highest rate was observed among carbapenem-susceptible *K. pneumoniae* isolates in 2018. 

Among patients with VAPs/VAEs, the SBSI rate was 11.6% (3,312/28,575) in males and 12.3% (2,474/20,099) in females (p=0.016). The mean number of hospital beds for patients who developed SBSI was 715.0±433.4 and 642.9±443.5 for those who did not (p<0.001).

While there was an increasing trend in SBSI rates during the years of observation within hospital types (except private hospitals), the highest number of VAPs/VAEs was in university hospitals. SBSI rates according to hospital type differed, with the highest rate found in training and research hospitals (14.6%) and the lowest rate in private hospitals (7.9%) (Table 1 (Tab. 1)).

Although the lowest number of VAPs/VAEs occurred in emergency ICUs (among the ICU types), they had the highest SBSI rate (17.9%). While anesthesiology and reanimation ICUs and surgical ICUs shared second place, the lowest rate (8.3%) was found in pediatric ICUs, which had the lowest numbers after emergency ICUs in terms of VAPs/VAEs (Table 1 (Tab. 1)).

### CAUTIs

Among the CAUTIs, the most common microorganism was *E. coli* with a rate of 37.9%, followed by *K. pneumoniae* (28.9%), *P. aeruginosa* (18.9%), and *A. baumannii* (14.3%). Of all bacterial strains investigated here, carbapenem resistance was the highest in *A. baumannii* isolates (69.4%; 95%CI=67.9%–71.0%). *K. pneumoniae* had the second highest rate (41.5%; 95%CI=40.3%–42.7%), followed by *P. aeruginosa* (31.5%; 95%CI=30.1%–32.9%) and finally *E. coli* (6.6%; 95%CI=6.1%–7.1%). ESBL production was higher in *K. pneumoniae* isolates (49.5%; 95%CI=48.3%–50.7%) than in *E. coli* isolates (42.6%; 95%CI=41.6%–43.7%) (p<0.001).

The highest SBSI rate for CAUTIs during the observation period was found for *K. pneumoniae* with a rate of 15.5%, and *A. baumannii* ranked second with 12.4%. The SBSI rate was similar in CAUTIs in which *E. coli* and *P. aureginosa* were the causative agents (Table 1 (Tab. 1)).

While the number of CAUTIs caused by *E. coli* and *A. baumannii* decreased year after year, the number of CAUTIs caused by *K. pneumoniae* increased. The SBSI rates increased significantly over the years in CAUTIs caused by *K. pneumoniae* and *A. baumannii* (Table 1 (Tab. 1)).

**Figure 3:**
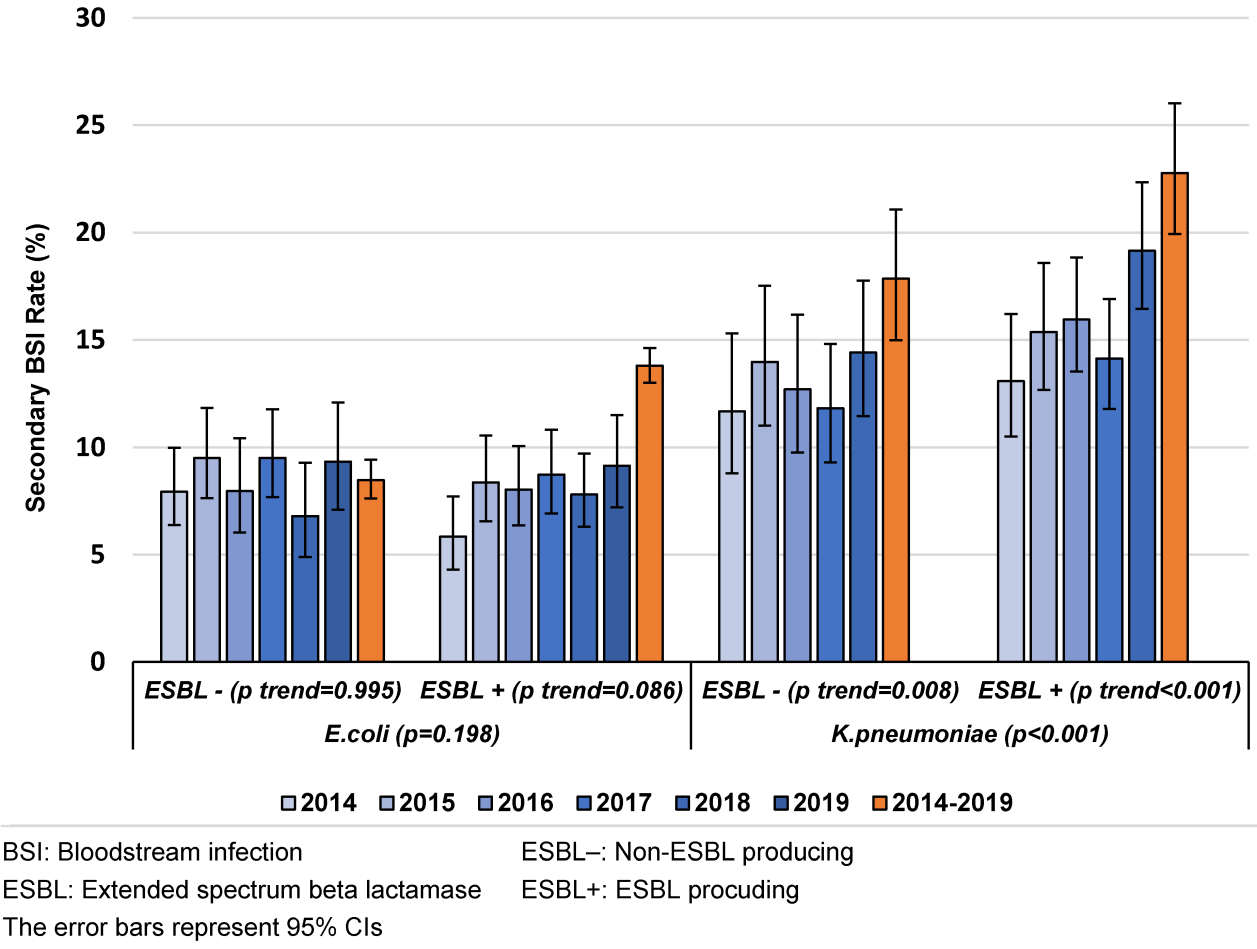
Secondary BSI rates in CAUTIs caused by *E. coli* and *K. pneumoniae* by ESBL production and years, Turkey, 2014–2019 (n=15,553)

Production of ESBL was found to be a risk factor for SBSI from CAUTIs caused by *K. pneumoniae*. While ESBL-producing and non-ESBL-producing *K. pneumoniae* had a higher SBSI rate than ESBL-producing and non-ESBL-producing *E. coli* (p<0.001 and p<0.001, respectively) in all years, an increasing trend of SBSI rates was observed in ESBL-producing and non-ESBL-producing *K. pneumoniae* with time (Figure 3 (Fig. 3)).

**Figure 4:**
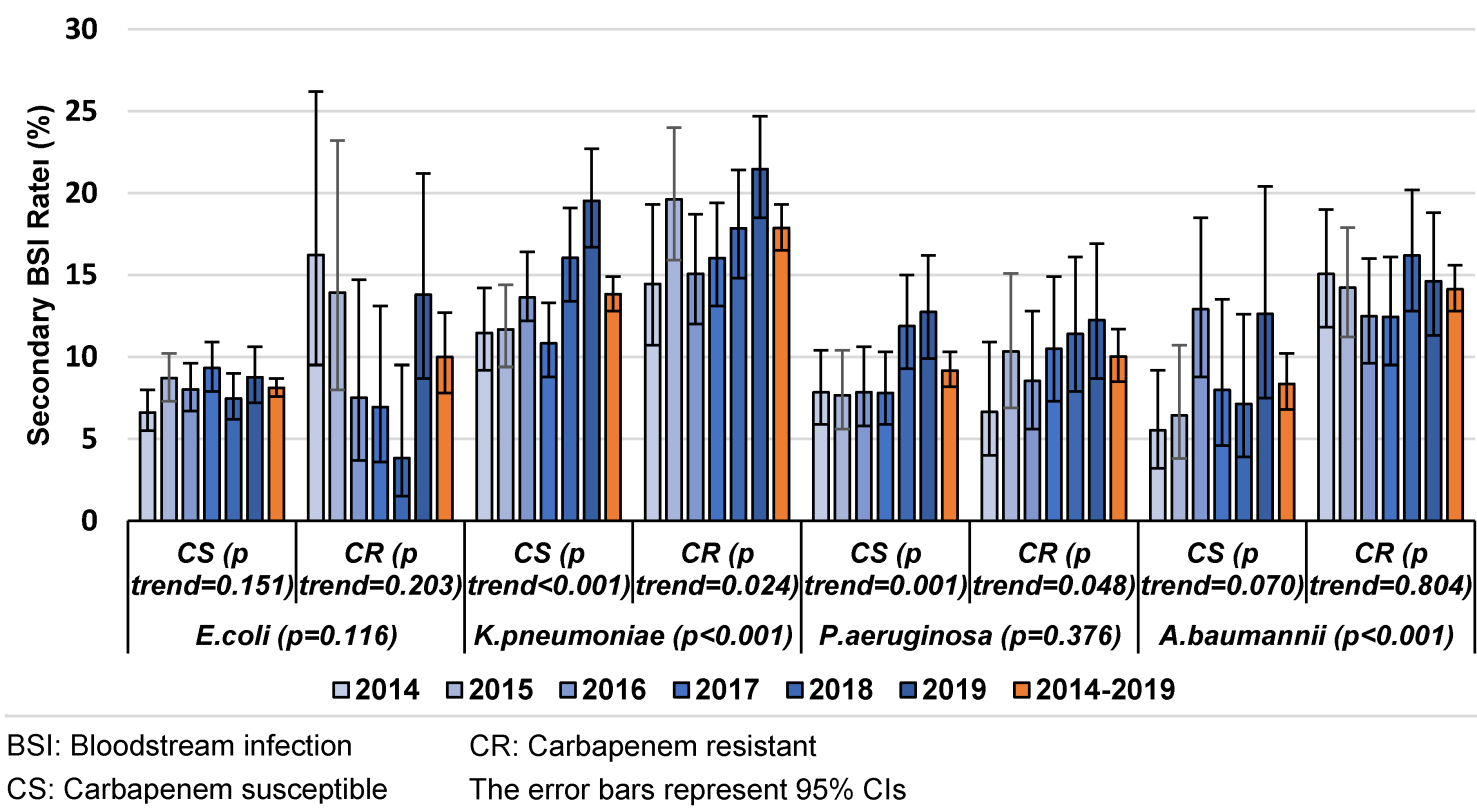
The distribution of secondary BSI rates in CAUTIs caused by different microorganisms, carbapenem resistance and years, Turkey, 2014–2019 (n=23,281)

Carbapenem resistance was found to be an other risk factor for SBSI in CAUTIs caused by *K. pneumoniae* and *A. baumannii*. The SBSI rates showed an increasing trend for carbapenem-susceptible and carbapenem-resistant *K. pneumoniae* and *P. aeruginosa* over time. In 2019, the rate reached 20% in the presence of carbapenem resistance for* K. pneumoniae*, and the highest rate was observed among carbapenem-susceptible *K. pneumoniae* isolates (Figure 4 (Fig. 4)).

Among patients with CAUTI, the SBSI rate was 10.1% (1,249/12,312) in males and 12.3% (1,349/10,969) in females (p<0.001). The mean number of hospital beds for those who developed SBSI was 679.2±435.1 and 625.7±425.6 for those that did not (p=0.007).

While SBSI rates showed an increasing trend with time within all hospital types (except private hospitals), the highest number of CAUTIs occurred in university hospitals. The highest SBSI rates were found in the training and research hospitals (12.6%) and university hospitals (11.6%). The lowest rate occurred in private hospitals (8.7%) (Table 1 (Tab. 1)).

Although the lowest number of CAUTIs occurred in emergency ICUs vs other ICU types, they had the highest SBSI rate (14.0%). This was followed by anesthesiology and reanimation ICUs (13.2%), surgical ICUs (12.6%), and finnally the lowest rate (7.7%) in pediatric ICUs, which had the lowest number after emergency ICUs in terms of CAUTIs (Table 1 (Tab. 1)).

### Multivariate analysis

In the multivariate analysis, microorganism type, ICU type, bed capasity and carbapenem resistance were risk factors for the development of SBSI for both types of infection. For VAPs/VAEs, female gender and hospital type were also identified as risk factors. The highest risk among microorganisms for both infections was found in *K. pneumoniae* isolates. Among ICU types, the highest risk for both infections was in emergency ICUs, while the lowest risk was found for pediatric ICUs. In VAPs/VAEs, the risk was 10.2% higher in females than in males. The risk increased by 24.8% in CAUTIs for hospitals with 750 and more than 750 beds, and by 19.0% in VAPs/VAEs. Among the hospitals, the highest risk in VAPs/VAEs was found in the training and research hospitals, followed by university hospitals and government hospitals (Table 2 (Tab. 2)). 

The effect of ESBL production for *K. pneumoniae* and *E. coli* was not found to be a risk factor in CAUTIs when adjusted for the effect of risk factors determined by the multivariate logistic regression analysis. In VAPs/VAEs, ESBL production increased the risk by 14.8%.

## Discussion

In this study, the SBSI rate in VAPs/VAEs and CAUTIs due to the four most common microorganisms in ICUs was 11.7%. The SBSI rates in all healthcare-associated pneumonias and urinary tract infections that developed in hospitalized patients in Turkey were 12.1 and 14.6 in 2019 and 2020, respectively [3], [7]. In this study, a significant increase was found in the SBSI rates for both types of infections between 2014 and 2019. Considering the surveillance data since 2007, there has been a significant decrease in invasive device-associated infections in ICUs throughout the country, and this increasing trend in SBSIs is striking [8].

The rate of SBSIs among all BSIs in hospitals has been reported to be up to 45% [2], [9], [10]. In Turkey, this rate was 16.7% and 16.8% in 2019 and 2020, respectively. The adjusted standardized infection rates calculated at the national level in 2017 proved that Turkey achieved success in the implementation of infection control measures in the ICUs for CLABSI and CAUTI. However, in this study, an increasing trend was found in the SBSI rates between 2014 and 2019 [11]. This increase may be due to the misclassification of primary and secondary BSIs. However, an evaluation of surveillance data in the United States showed that there was no significant misclassification between primary and secondary BSIs [12]. If the reduction in primary BSI is not real, this may be explained by more blood culture collection in those with primary infections and better investigation of the primary source of infection in patients with a positive blood culture. However, together with the total number of primary BSI, the total number of BSIs and even the number of SBSIs are also decreasing in Turkey. Therefore, the probability of misclassification seems slight. Less reduction in SBSI than in primary BSI may have resulted in a proportional increase in secondary BSI among all BSIs.

The most common sources of SBSI were reported to be lower respiratory tract infections, urinary tract infections and surgical site infections (SSIs), respectively. In various other studies, however, the type of primary infection that caused SBSI is in different order [2], [13], [14], [15], [16]. As this study was conducted in ICUs, SSIs were not evaluated. However, SSIs in Turkey lag behind, with an SBSI rate of 4.0% in 2020 [3]. The variation among studies may be due to differences in healthcare systems and patient populations, as well as methodological differences. For example, a study found that up to 10% of SBSIs were due to some invasive procedure, such as retrograde endoscopic cholangiopancreatography and percutaneous transhepatic cholangiogram [2].

Among the microorganisms examined, the highest risk for the development of SBSI was presented by *K. pneumoniae* in both VAPs/VAEs and CAUTIs. While the risk increases by 41.8% in VAPs/VAEs caused by *K. pneumoniae*, it increases by 82.6% in CAUTIs. Infections caused by *Klebsiella* spp. are responsible for approximately one-fifth of healthcare-associated infections reported in Turkey in 2020 [3]. It is the second most common agent after *A. baumannii* in VAPs/VAEs, and is also the second most common agent in CAUTIs after *E. coli*. In this respect, the burden of BSIs secondary to infections with *K. pneumoniae* on the health system can be considered to be quite high.

The number of emergency ICUs is lower in Turkey compared to the other ICU types, and patient-days, average length of stay, device utilization rate and infection rates are much lower in emergency ICUs [17]. However, in our study, the ICU type with the highest risk of developing SBSI for both types of infection was emergency ICUs. The risk was also greater in all the other ICU categories compared to pediatric ICUs, so the burden for SBSI is much higher in the other ICUs, where patient-days and the number of infections are much higher than in emergency ICUs.

It is expected that the risk is higher in hospitals with a bed capacity of 750 or more, because these hospitals are mostly provide tertiary care and serve patients with more complicated needs. The fact that the risk of VAPs/VAEs is higher in women than in men may be due to the effect of other confounding factors, such as age, which could not be evaluated in this study. 

The finding that the risk is higher for VAPs/vAEs in the traning/research hospitals and university hospitals is not unexpected, because these hospitals are tertiary hospitals and have higher infection rates compared to the others [17]. In addition, the laboratory capacities and surveillance responsiveness of the hospitals may be better. However, hospital type has not been identified as a risk factor for CAUTIs. This may be because the use of urinary catheters is more common, and there is less heterogeneity in urinary catheter care between hospitals than in ventilator use. The national reports show that the difference among hospital types for CAUTIs is less than for VAPs.

The presence of carbapenem resistance and ESBL production of the microorganism causing the infection increases the risk of SBSI. Possible causes of these infections with resistant microorganisms are more difficult treatment or delayed initiation of appropriate treatment. The fact that carbapenem resistance and ESBL production are a serious problem in Turkey may contribute significantly to the development of SBSI. Therefore, monitoring the epidemiology of bloodstream infections and antimicrobial resistance at the regional and hospital level, and even at the ICU level, would be beneficial for timely initiation of appropriate treatment. In addition, control of the primary source of infection may reduce treatment failure in SBSI. If BSI is primary, only antimicrobial therapy may be sufficient, but it may be insufficient when BSI is secondary to another infection. For example, surgical interventions may be necessary in BSI secondary to abscesses, soft tissue infections, and infections in intra-abdominal organs or cavities [4]. In a study in Turkey, one of the independent factors related to mortality in patients with CAUTIS in ICUs was the development of secondary bloodstream infection [18].

Some limitations should be considered when interpreting the results. Although data were collected mostly by certified infection control nurses, classification errors may have occurred due to the retrospective nature of the study. Nevertheless, there may be some differences in surveillance and laboratory capacity among the hospitals. Nevertheless, the resistance data were compatible with the National Antimirobial Resistance Surveillance System. Some other factors such as colistin resistance and multidrug resistance may also be confounding factors for the effect of carbapenem resistance and ESBL production on SBSI. In addition, because the clinical signs and symptoms of the patients could not be evaluated, the diagnosis of VAP, VAE, CAUTI and SBSI was not validated. The culturiing routines of the hospitals may have affected the diagnoses. Factors such as age, underlying diseases, and length of stay were not addressed in the study. Variables such as hospital type and ICU type were included in the study as surrogate variables for the comparability of the patient population. Since two types of infections and four agents in ICUs were examined, the findings cannot be generalized to wards, other infections or all agents.

Despite the limitations, the study covers a period of six years and all hospitals in Turkey. The data was collected by infection-control professionals trained for surveillance and recorded electronically. The sample size is large enough to allow interferences based on the data.

## Conclusions

Studies to date have generally examined patient-level risk factors, such as underlying diseases, previous hospitalization, previous antibiotic therapy, and invasive interventions for primary and secondary BSIs [19], [20], [21], [22], [23], [24]. In this study, hospital-level data were emphasized. It would be expeditious to recognize primary infections earlier and to distinguish SBSIs from primary BSIs by determining higher-risk hospitals, higher-risk areas in hospitals, infections with more dangerous bacteria and bacteria with problematic antibiotic-resistance patterns. Thus, morbidity, mortality and cost due to SBSIs can be reduced. In conclusion, it is important to be alert to the development of SBSI, especially in patients with CAUTIs or VAPs/VAEs caused by carbapenem-resistant and/or ESBL-producing *K. pneumoniae*.

## Notes

### Competing interests

The authors declare that they have no competing interests.

### Acknowledgments

Thanks to all infection control nurses and physicians in all hospitals in Turkey for providing us this data.

## Figures and Tables

**Table 1:**
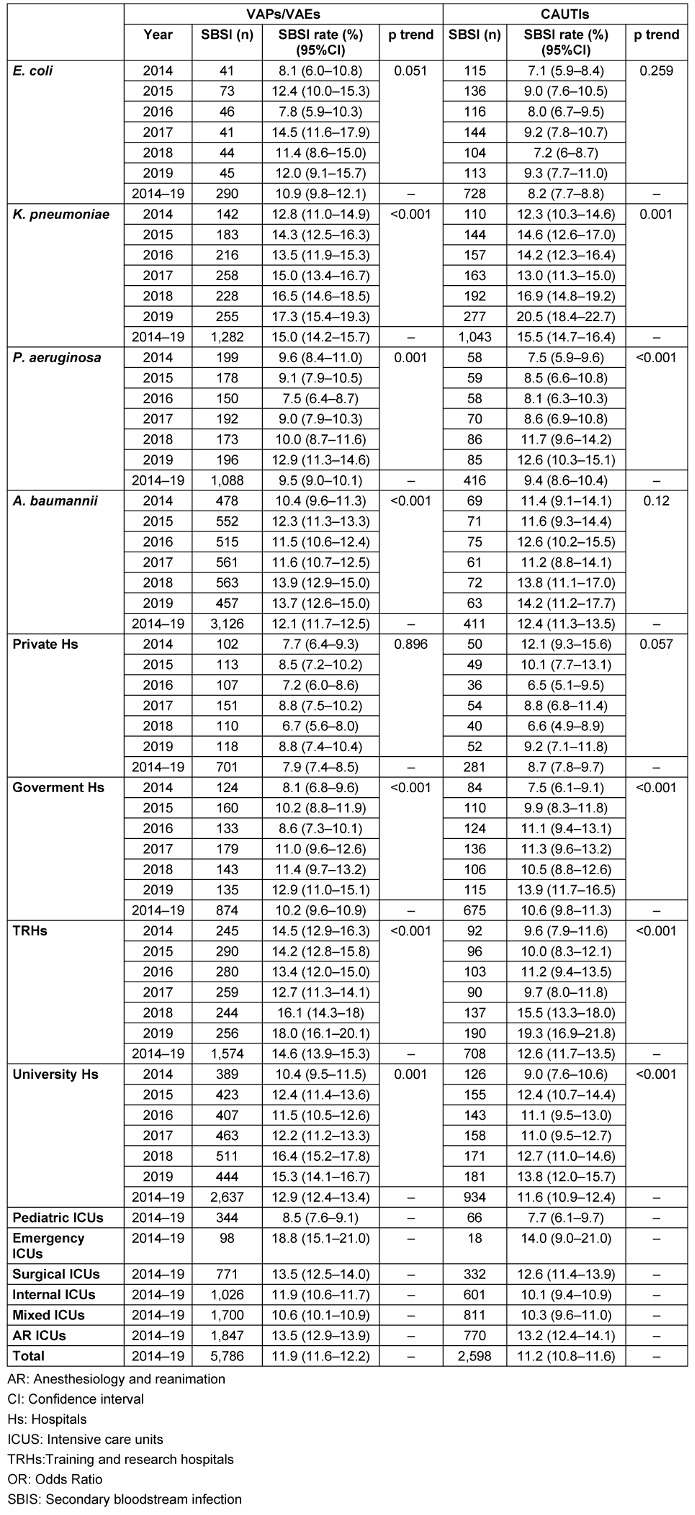
SBSI rates by variables stratified according to infection type, Turkey, 2014–2019, n=71,955 infections

**Table 2:**
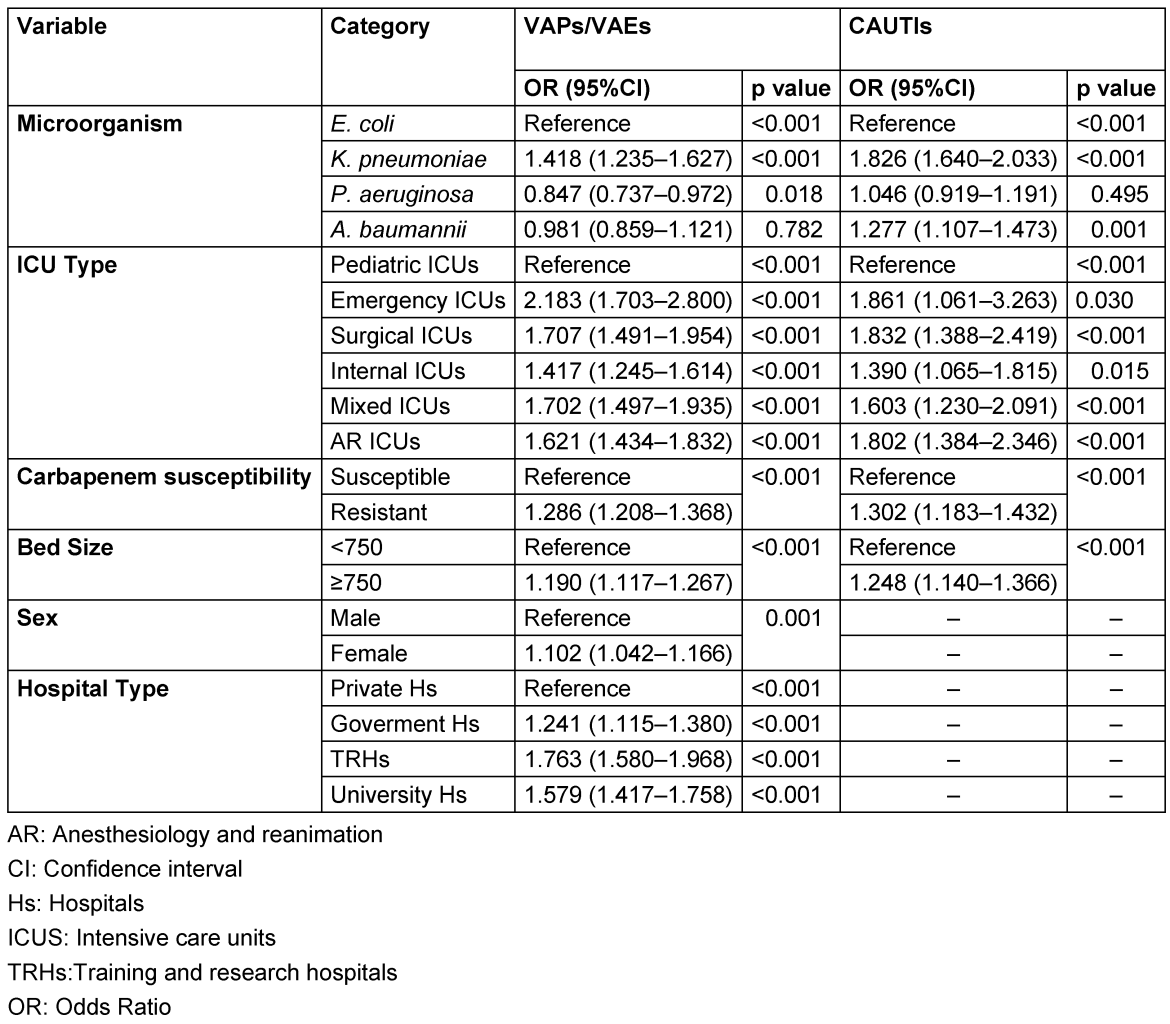
The multivariable analysis of variables independently associated with the development of BSI by infection type, Turkey, 2014–2019 (n=71,955)
